# CDX2 in colorectal cancer is an independent prognostic factor and regulated by promoter methylation and histone deacetylation in tumors of the serrated pathway

**DOI:** 10.1186/s13148-018-0548-2

**Published:** 2018-09-26

**Authors:** Janina Graule, Kristin Uth, Elia Fischer, Irene Centeno, José A. Galván, Micha Eichmann, Tilman T. Rau, Rupert Langer, Heather Dawson, Ulrich Nitsche, Peter Traeger, Martin D. Berger, Beat Schnüriger, Marion Hädrich, Peter Studer, Daniel Inderbitzin, Alessandro Lugli, Mario P. Tschan, Inti Zlobec

**Affiliations:** 1 Institute of Pathology, University of Bern, Murtenstrasse 31, Room L310, 3008 Bern, Switzerland; 2 Graduate School for Cellular and Biomedical Sciences, University of Bern, Freiestrasse 1, 3012 Bern, Switzerland; 3 Department of Surgery, Klinikum rechts der Isar, Technische Universität München, Ismaninger Strasse 22, Munich, 81675 Germany; 4 Careanesth AG, Nelkenstrasse 15, Zürich, 8006 Switzerland; 5 Department of Medical Oncology, University Hospital of Bern, 3010 Bern, Switzerland; 6 Division of Medical Oncology, Norris Comprehensive Cancer Center, Keck School of Medicine, University of Southern California, Los Angeles, 90033 CA USA; 7 Department of Visceral and Internal Medicine, University Hospital of Bern, 3008 Bern, Switzerland; 8 University of Bern and Bürgerspital Solothurn, Schöngrünstrasse 42, 4500 Solothurn, Switzerland

**Keywords:** Colorectal cancer, CDX2, Methylation, Histone modification, Prognosis, Biomarker

## Abstract

**Background:**

In colorectal cancer, CDX2 expression is lost in approximately 20% of cases and associated with poor outcome. Here, we aim to validate the clinical impact of CDX2 and investigate the role of promoter methylation and histone deacetylation in CDX2 repression and restoration.

**Methods:**

CDX2 immunohistochemistry was performed on multi-punch tissue microarrays (*n* = 637 patients). Promoter methylation and protein expression investigated on 11 colorectal cancer cell lines identified two CDX2 low expressors (SW620, COLO205) for treatment with decitabine (DNA methyltransferase inhibitor), trichostatin A (TSA) (general HDAC inhibitor), and LMK-235 (specific HDAC4 and HDAC5 inhibitor). RNA and protein levels were assessed. HDAC5 recruitment to the CDX2 gene promoter region was tested by chromatin immunoprecipitation.

**Results:**

Sixty percent of tumors showed focal CDX2 loss; 5% were negative. Reduced CDX2 was associated with lymph node metastasis (*p* = 0.0167), distant metastasis (*p* = 0.0123), and unfavorable survival (multivariate analysis: *p* = 0.0008; HR (95%CI) 0.922 (0.988–0.997)) as well as BRAF^V600E^, mismatch repair deficiency, and CpG island methylator phenotype. Decitabine treatment alone induced CDX2 RNA and protein with values from 2- to 25-fold. TSA treatment ± decitabine also led to successful restoration of RNA and/or protein. Treatment with LMK-235 alone had marked effects on RNA and protein levels, mainly in COLO205 cells that responded less to decitabine. Lastly, decitabine co-treatment was more effective than LMK-235 alone at restoring CDX2.

**Conclusion:**

CDX2 loss is an adverse prognostic factor and linked to molecular features of the serrated pathway. RNA/protein expression is restored in CDX2 low-expressing CRC cell lines by demethylation and HDAC inhibition. Importantly, our data underline HDAC4 and HDAC5 as new epigenetic CDX2 regulators that warrant further investigation.

**Electronic supplementary material:**

The online version of this article (10.1186/s13148-018-0548-2) contains supplementary material, which is available to authorized users.

## Background

CDX2 is a homeobox protein responsible for the maintenance of the intestinal phenotype [1, 2]. Over the last decade, CDX2 has been linked to colorectal cancer (CRC) progression, with reduced expression of the protein associated with more advanced tumor stage, vessel invasion, and metastasis [3–7]. Many studies, including the work by Dalerba et al., underline the unfavorable survival time in patients with a complete absence of CDX2 in the tumor [8], a feature that occurs in approximately 5% of patients [7, 8]. Furthermore, they demonstrate that CDX2-negative CRC patients may benefit from chemotherapy, particularly in a stage II setting [8].

Reduced CDX2 protein expression is related to certain molecular alterations during colorectal tumorigenesis. Previous work by our group and others shows that nearly all sporadic microsatellite unstable (MSI) cancers show some degree of loss of the protein in the tumor, whether in a small or substantial percentage of cells [3, 5, 9]. This loss is not however limited to MSI-high cancers, but is also found in microsatellite stable (MSS) tumors with BRAF mutation and high-level CpG island methylator phenotype (CIMP), in other words, in cancers deriving from the so-called serrated pathway [10]. More than 20% of CRCs show some degree (or complete loss) of CDX2 protein in the tumor, which is often reported along with a preponderance for female gender and right-sided tumor location, two features frequently associated with serrated lesions [11].

Since CDX2 mutations are extremely rare events in CRCs [12], we hypothesized that epigenetic changes, such as promoter hypermethylation or histone deacetylation could be responsible for significant downregulation or absence of CDX2, particularly in the group of tumors displaying “serrated” molecular features (BRAF mutation, MSI, and CIMP) [13]. In fact, human serrated adenomas with high-grade dysplasia have been shown to have significantly greater frequencies of CDX2 hypermethylation than other polyp types (like classical adenomas) [14].

In this study, we aim (1) to validate the clinical relevance of CDX2 in a large group of CRC patients (*n* = 637), (2) to determine whether epigenetic modifications contribute to CDX2 repression, and (3) to restore CDX2 expression in vitro by targeting methylation and histone deacetylation.

## Methods

### Patients

Two retrospective cohorts were investigated. Patient characteristics are found in Additional file 1: Table S1.

#### Cohort 1 (Germany)

The cohort initially included 341 primary resected colon cancer (no rectal cancer) patients treated at the Department of Surgery at the Technical University Munich (TUM) hospital, Munich, Germany, between 1993 and 2005. Clinical and pathological features for this cohort included age at diagnosis, gender, tumor location, TNM stage (UICC 6th ed.), R classification, and tumor grade. After exclusion of patients with unavailable tumor material for this study, the final cohort comprised 252 patients of which 237 (94%) had information on therapy and survival. Overall 5-year survival rate was 66.6%.

#### Cohort 2 (Switzerland)

This cohort encompasses 385 surgically treated CRC patients. Clinical features retrieved from patient charts were age at diagnosis, tumor location, and gender. Survival information, follow-up, and therapy information were available for 286 (82.9%). Histopathology was re-reviewed according to the TNM 7th edition and is summarized in Additional file 1: Table S1. MSI status determined by PCR was available for 128 patients. Adjuvant treatment for high-risk stage II and stage III colon cancer consisted of a 5-FU or capecitabine-based chemotherapy (5-FU orcapecitabine ± oxaliplatin). Palliative first-line chemotherapy for stage IV patients comprised either the FOLFOX, XELOX, or FOLFIRI regimen with or without bevacizumab/cetuximab, while anti-EGFR treatment was applied only to KRAS wild-type patients. Overall 5-year survival was 60.7%.

### Ethics, consent, and permissions

The ethics committees of the Klinikum rechts der Isar and Canton of Bern approved the use of data and patient material for this study (nos. 1926/7 and 200/2014, respectively).

### Next-generation tissue microarray (ngTMA®) construction

Tissues from all 637 patients were retrieved from the corresponding archives of the Institutes of Pathology at the TUM and University of Bern. One to two H&E slides were sectioned from each block, and the slides were scanned (P250 Flash II, 3DHistech, Hungary). Digital slides were annotated using a tissue microarray tool by placing six to eight different circles onto various histological areas (Additional file 1: Figure S1) [15]. The annotated digital slide was then aligned with the tumor block and cored using a 0.6-mm-diameter TMA tool (TMA Grandmaster, 3DHistech, Hungary). In addition, the TMA tool of 1.0 mm diameter was used to punch out cores from cohort 1, which were placed into tubes for downstream molecular analysis.

### Cell lines and treatment

CRC cell lines (LS174T, T84, LS180, HCT15, HT29, SW620, COLO205, HCT116, COLO320, LoVo, CaCo2) were obtained from the American Type Culture Collection (ATCC, Manassas, VA, USA) and grown in media with supplements as described in Additional file 1: Table S2 under humidified atmosphere of 5% CO2.

Treatment with DNA methyltransferase inhibitor (DNMTi): 3.5 × 10^4^ COLO205 or SW620 cells were seeded in six-well plates and treated with 0.5% DMSO, 1.25 μM, 2.5 μM, 5 μM, and 10 μM of decitabine (Stock 50 mM in DMSO, Cat.#S1200, Selleckchem, Houston, TX, USA) for 48 h.

Treatment with histone deacetylase inhibitors (HDACi): 3.5 × 10^4^ COLO205 or SW620 cells were seeded in six-well plates and treated with 0.01% DMSO, 50 nM trichostatin A (Stock 5 mM in DMSO, Cat.# T8552, Sigma-Aldrich, St. Louis, MO, USA) and 20 nM LMK-235 (Stock 10 mM in DMSO, Cat.# S7569, Selleckchem) alone or in combination with 2.5 μM, 5 μM, and 10 μM of decitabine for 48 h.

3.5 × 10^4^ HT29, SW620, LS174T, and LoVo cells were seeded in six-well plates and treated with 8 × 10^−3^ DMSO, 5 nM, 10 nM, 20 nM, 40 nM, and 80 nM of LMK-235 for 48 h.

### RNA extraction and real-time quantitative RT-PCR (qPCR)

RNA was extracted using the miRCURY RNA Isolation Kit (Prod.#300110; Exiqon, Vedbaek, Denmark) according to the manufacturer’s instructions. RNA concentrations were measured using NanoDrop (Thermo Scientific) and adjusted to 500 ng/10 μL. cDNA Synthesis Reagent (5xRT Super Mix, Cat. #B24403; Biotool, Houston, TX, USA) was added to the diluted RNA, RT-PCR performed using a Veriti 96-well Thermal Cycler (Model #9902; Applied Biosystems, Rotkreuz, Switzerland), and H_2_O added to an final concentration of 10 ng/μL cDNA. qPCR was performed using 10 ng/μL cDNA and TaqMan Fast Universal PCR Master Mix (Applied Biosystems). For quantification of *CDX2* and *HIC1* mRNA, the Taqman® Gene Expression Assay Hs01078080_m1 and Hs00359611_s1 (both Applied Biosystems), respectively, was used. *HMBS* primers and probes have been described earlier [16]. Raw Ct values were normalized to *HMBS* and to the untreated controls and are shown as *n*-fold changes (2 ^−ΔΔCt^ analysis).

### Western blot analysis

Cell lysates were prepared using a buffer containing 8 mM urea, 0.5% Triton-X, and proteinase inhibitors (25× PIC Complete, Rosch), protein concentration determined with Bradford Assay (Bio-Red Protein Assay; BioRad, Cressier, Switzerland) and 10 μg samples, mixed with loading buffer (4× Laemmli Sample Buffer, Cat: #161-0747, BioRad) and loaded on Mini-Protean TGX Stain Free Gels (12%, 15-well, Cat. #456-8095; BioRad). After UV activation, proteins were transferred to a PVDF membrane (0.2 μm PVDF, Cat. #170-4156; BioRad) using a Trans-Blot Turbo Transfer Pack (Mini format; BioRad). Membranes were blocked with 5% TBS-milk for at least 45 min. Blots were incubated with anti-CDX2 (1:500 in BSA, EPR2764Y—28.8 μg/mL, Rabbit Monoclonal, Cell Marque; Sigma-Aldrich) over night at 4 °C followed by incubation with anti-rabbit (1:10,000 in milk; Cell Signalling Technology, Leiden, The Netherlands) for at least 2 h at RT. Detection took place using ECL (Clarity Western ECL Substrate, Cat. #170-5060; BioRad) and ChemiDoc (MP, Serial #731BR00765; BioRad). Quantification was performed with ImageJ.

### Chromatin immunoprecipitation (ChIP)

HEK-293T cells have been transiently transfected using calcium phosphate [17] and 2 μg of FLAG-HDAC5 (Addgene #32213) expression plasmid in a 10-cm dish. After 48 h, cells were harvested and processed for ChIP using the ChIP-IT Express Chromatin Immunoprecipitation Kit (ChIP-IT Express, Active Motif, Carlsbad, CA, USA) according to the manufacturer’s recommendations. In parallel, FLAG-HDAC5 expression was determined by Western blotting (data not shown). For immunoprecipitation, 2.5 μg anti-Flag antibody (Sigma, Cat.#F3165) was used. Antibodies against acetyl-histone H3 (Cell Signalling, Cat.#9715) and mouse IgG (PP64B, Upstate, Millipore) served as positive and negative controls, respectively. PCR was performed using JumpStart *Taq* (Sigma-Aldrich) and the following primers, specifically selected to cover a 2500-bp genomic region upstream of the transcription start site (TSS) of the *CDX2* gene: 2000–2500 bp, F: 5′-CTTTCCATGGCTGGAGCACT-3′, R: 5′-CGCTGGCTAATTGTCCCTGT-3′; 1500–2000 bp, F: 5′-CATTCCCACCCCATCAGGTC-3′, R: 5′-CCAAGGAGCTGTGCACTCAA-3′; 1000–1500 bp, F: 5′-ACAGACAAGTGCAGGTCTCC-3′, R: 5′-CCCAGCTCGGTTTCAGCA-3′; and TSS–500 bp, F: 5′-TGGAGGTTAAAGTGCACCAGGT-3′, R: 5′-GACACCAATGGTTGGAGACG-3′. As a positive control for HDAC5 recruitment, we amplified a genomic region of the HDAC5 repressed fibroblast growth factor 2 (*FGF2*) gene using the following published primers: F: 5′-TGGAGGTTAAAGTGCACCAGGT-3′ and R: 5′-GACACCAATGGTTGGAGACG-3′ [18].

### DNA extraction and CDX2 methylation analysis

Genomic DNA was extracted from selected tumoral area of FFPE tissues using QIAamp DNA FFPE Tissue Kit (Qiagen; Hilden, Germany). Bisulfite conversion and pyrosequencing were used to analyze *CDX2* methylation in two different promoter regions. Both regions are located on chromosome 13, GRCh38.p7 Primary Assembly (NC_000013), region 1: 27970684-27970645 and region 2: 27970508-27970478. PCR conditions and details on primer sequences and region to analyze are outlined in Additional file 1: Methods.

### MS-MLPA for CIMP status

Methylation-specific multiplex ligation-dependent probe amplification (MS-MLPA) was performed according to standard protocol for CIMP status evaluation and *BRAF*^V600E^ mutation. Promoter methylation of *CACNA1G*, *IGF2*, *NEUROG1*, *RUNX3*, *SOCS1*, *CDKN2A*, *MLH1*, and *CRABP1* was analyzed by SALSA MLPA probemix ME042-C1 (MRC Holland, Amsterdam, Netherlands). A gene was considered methylated when one fourth (25%) or more probes were at least 30% methylated. This cutoff was set as it corresponds with the highest background methylation value in the healthy tissue control.

### Immunohistochemistry and in situ hybridization

All ngTMA blocks were sectioned at 2.5 μm, and immunohistochemistry was performed on an automated immunostainer (Leica Bond Rx or Ventana Benchmark Ultra) for CDX2 (clone ERP2764Y, Cell Marque, 1:400, Tris 95° 30′), *BRAF*^V600E^ protein (clone VE1, Roche, CC1 99° 72′), and MLH1 (clone ES05, Leica Novocastra, 1:200, Tris 95° 30′). CDX2 and MLH1 protein expression was evaluated by estimating the number of immunoreactive nuclei in each tumor punch, then an average positive count across all cores from the same patient lead to the final marker value for statistical analysis. Since CDX2 is normally present in all cells of the normal colonic mucosa, we quantified the percentage of immunoreactive cells in the tumor then defined a “reduced or loss of” expression when less than 100% of cells were stained and a complete loss of expression when 0% of cells where stained. VE1 was scored as positive or negative. Any doubt regarding positive staining of VE1 was confirmed by pyrosequencing. RNA expression was evaluated semi-quantitatively [19] across all tumor punches in cohort 1 using RNAscope 2.0 FFPE assay and probes for *CDX2*, the bacterial gene *dapB*, as negative control and the housekeeping gene *PPIB* as positive control (Advanced Cell Diagnostics, Inc.). Scoring was performed as previously described [17]. Briefly, score 0 = no staining, score 1 = difficult to see under 40x, score 2 = difficult to see under 20x but easy under 40x, score 3 = difficult to see under 10x but easy under 20x, and score 4 = easy to see under 10x.

Immunohistochemistry was also performed on cell lines. 1 × 10^6^ cells of COLO205 and SW620 cell lines were seeded, treated with decitabine and/or TSA or LMK-235 and harvested using trypsin after 48 h and 72 h. Cells were washed with PBS, formalin-fixed and paraffin embedded (FFPE), and immunohistochemically stained for CDX2. Slides were scanned with a Pannoramic 250 Flash II (3DHistech Ltd., Budapest, Hungary). Cell quantification was performed with the open source image analysis software QuPath [20], using watershed cell detection on optical density sum images and subsequent random trees classification of the detected cells.

### Statistics

Descriptive statistics, non-parametric Wilcoxon rank sum test or chi-square tests were used to analyze differences in CDX2 staining with categorical features. Survival time analysis was performed using both log-rank tests and Kaplan-Meier curves and Cox proportional hazards regression models in multivariable analysis, adjusting for potential confounding factors. Hazard ratios and 95%CI were used to determine the effect differences. Spearman correlation coefficients were calculated to determine the strength of relationship between methylation and protein expression. Student’s *t* test was used to compare mean methylation percentage in CDX2-positive or CDX2-negative cell lines. For statistical analysis of four biological replicates of qPCR and Western blot results, Mann-Whitney test was performed. *p* values < 0.05 were considered statistically significant. No adjustment for multiple comparisons was performed [21]. Analyses were performed using SAS V9.3 (The SAS Institute, Cary, NC) and PRISM, GraphPad Software.

## Results

### Distribution of CDX2 protein expression scores

CDX2 protein expression ranged from 0 to 100%. Additional file 1: Figure S2 highlights the distribution of expression from cohort 2 (A) as well as representative immunostaining (B-D). Thirty-nine patients (5.0%) showed a complete absence of CDX2 protein in the tumor. The percentage of CDX2 immunostained tumor cells was 66% on average, with a median of 78.8%. In terms of different tumor areas, there was no difference in expression between the tumor center and invasion front; however, tumor budding cells were frequently seen with an absent CDX2 staining.

### Relationship between mRNA ISH scores and protein expression

One thousand four hundred sixty-one punches were evaluated for mRNA ISH with corresponding protein data. There was a strong and statistically significant correlation between the CDX2 protein expression scores in the tumors and the corresponding mRNA ISH scores (*r* = 0.99, *p* < 0.0001) indicating that RNA expression and protein expression were highly associated. The mean percentage of CDX2 protein expression across all tumors was 44.2% (score 0), 52.7% (score 1), 65.8% (score 2), 76.2% (score 3), and 90.8% (score 4) (*p* < 0.0001) (Additional file 1: Figure S3).

### Clinicopathological features associated with progressive CDX2 loss

In cohort 1, there was a significant correlation between reduced CDX2 expression and female gender (*p* = 0.0338); more advanced pT classification (*p* = 0.0068), lymph node metastasis (*p* = 0.0167), and distant metastasis (*p* = 0.0123); and higher tumor grade (*p* = 0.0163) (Table 1). Similar correlations could be found for cohort 2 with significant associations between reduced CDX2 and histological subtype (*p* = 0.009), right-sided tumor location (*p* = 0.0135), more advance pT stage (*p* = 0.0002), distant metastasis (*p* = 0.0337), higher tumor grade (*p* = 0.0004), lymphatic vessel invasion (*p* = 0.00136), and a trend to venous vessel invasion (*p* = 0.0706).

**Table 1:** Association of progressive CDX2 loss with clinicopathological features in two cohorts

Clinicopathological feature	COHORT 1 (*n* = 252)		COHORT 2 (*n* = 385)	
	CDX2% (mean/median)	*p* value	CDX2% (mean/median)	*p* value
Gender				
Male	61.5/71.7	0.0338	72.6/85	0.5675
Female	52.6/56.7		67.6/83.1	
Histological subtype				
Adeno	n/a		71.9/85	0.009
Mucinous	n/a		66.6/75	
Other	n/a		41.9/51.3	
Tumor location				
Left	58.2/60.8	0.812	69.5/86.3	0.0135
Right	57.3/65.8		75.6/90	
Rectum	–		68.1/75	
pT				
pT1	62.3/75.0	0.0068	83.8/93.8	0.002
pT2	55.8/63.3		68.7/82.5	
pT3	55.9/64.5		73.7/84.4	
pT4	37.0/26.7		56.3/66.7	
pN				
pN0	61.6/71.7	0.0167	72.5/86.3	0.1891
pN1-2	52.6/58.8		69.0/82.5	
pM				
pM0 (c)	65.2/76.7	0.0123	72.5/85	0.0337
pM1-2	48.9/60		60.2/71.3	
Tumor grade				
G1-2	65.4/76.7	0.0163	74.8/85	0.0004
G3	47.0/40.0		57.2/70.4	
Lymphatic invasion				
L0	n/a	–	74.7/88.8	0.0136
L1	n/a		68.9/75.8	
Venous invasion				
V0	n/a	–	74.2/84.4	0.0706
V1	n/a		67.7/80	
Perineural invasion				
Pn0	n/a	–	70.0/80	0.7978
Pn1	n/a		69.8/85	
BRAF				
Wild-type	59.6/66.7	0.0044	76.0/87.5	< 0.0001
Mutated	43.0/38.3		26.4/4.4	
MMR status				
Deficient	48.1/47.5	0.0077	43.5/50	0.0005
Proficient	61.8/71.7		69.5/79.4	

*n/a* not available

### CDX2 is an adverse and independent prognostic factor

**Fig. 1:**
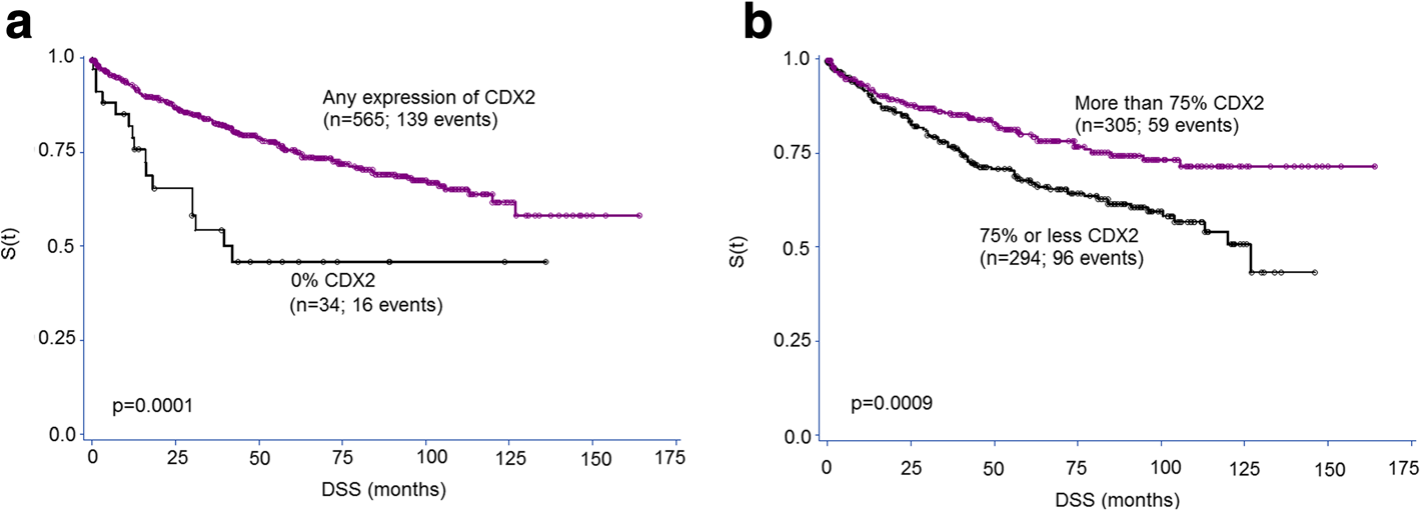
Kaplan-Meier survival curves showing survival time differences using two different cutoff values for CDX2. **a** Complete absence of staining (0%). **b** Loss of CDX2 with less than 75% staining. Log-rank test

Survival analysis was performed on the combined set of patients; 599 patients were available for analysis. In univariate analysis, reduced CDX2 expression was significantly related to worse overall survival (Cox regression analysis using percentage of positive cells) (*p* = 0.0008; HR (95%CI) 0.992 (0.988–0.997)). The survival effect of CDX2 was also evaluated using two different cutoff values, found in the literature: 0% (versus any expression) [8] and a threshold of 75% (focal versus diffuse) [10]. Thirty-four of 599 patients had tumors with 0% expression, and 16 (47.1%) died over the course of follow-up (Fig. 1).

Five hundred and sixty-five patients had tumors with any CDX2 expression, and 139 died during follow-up. In both instances, there was a significant and marked effect of CDX2 absence/loss on survival. However, of the two cutoffs interrogated, only the 0% cutoff was found to have an independent prognostic effect on outcome, after adjusting for TNM stage and postoperative therapy (Table 2). Our analysis of CDX2-negative patients with and without chemotherapy shows no difference in the overall survival with postoperative treatment. However, due to low statistical power of the negative subgroup, we cannot adequately evaluate the survival benefit with chemotherapy here.

**Table 2:** Multivariable analysis of CDX2 (absence versus any positive expression) adjusting for TNM stage and postoperative therapy

	HR (95%CI)	*p* value
CDX2		
Negative	1.0	
Positive	0.35 (0.17–0.71)	0.0037
TNM stage		
TNM I (vs IV)	8.85 (4.05–19.2)	< 0.0001
TNM II (vs IV)	6.33 (3.65–10.9)	< 0.0001
TNM III (vs IV)	3.62 (2.18–6.02)	< 0.0001
Postoperative therapy		
None	1.0	
Treated	1.01 (0.83–1.23)	0.9172

### CDX2 loss is associated with molecular features of the serrated pathway

*BRAF*^V600E^ mutation was found in 10.4% of patients, while mismatch repair (MMR) deficiency in 12.2% of all patients in both cohorts. Expression of CDX2 was significantly reduced in tumors with *BRAF*^V600E^ mutations (*p* = 0.0044 cohort 1; *p* < 0.001 cohort 2) and tumors with defective MMR (*p* = 0.0077 cohort 1; *p* = 0.0005 cohort 2). Additional file 1: Figure S4 outlines the progressive loss of CDX2 protein with changes in both MMR status (proficient or deficient) and BRAF status (wild-type or mutation) across both cohorts (*n* = 590). In comparison to MMR-proficient/BRAF WT tumors (70.1% CDX2), those with MMR-deficient/*BRAF*^V600E^-mutated cancers (29.3% CDX2) have a significantly reduced expression (*p* < 0.0001).

### *CDX2* promoter methylation is a mechanism of protein loss in CRC cell lines

**Fig. 2:**
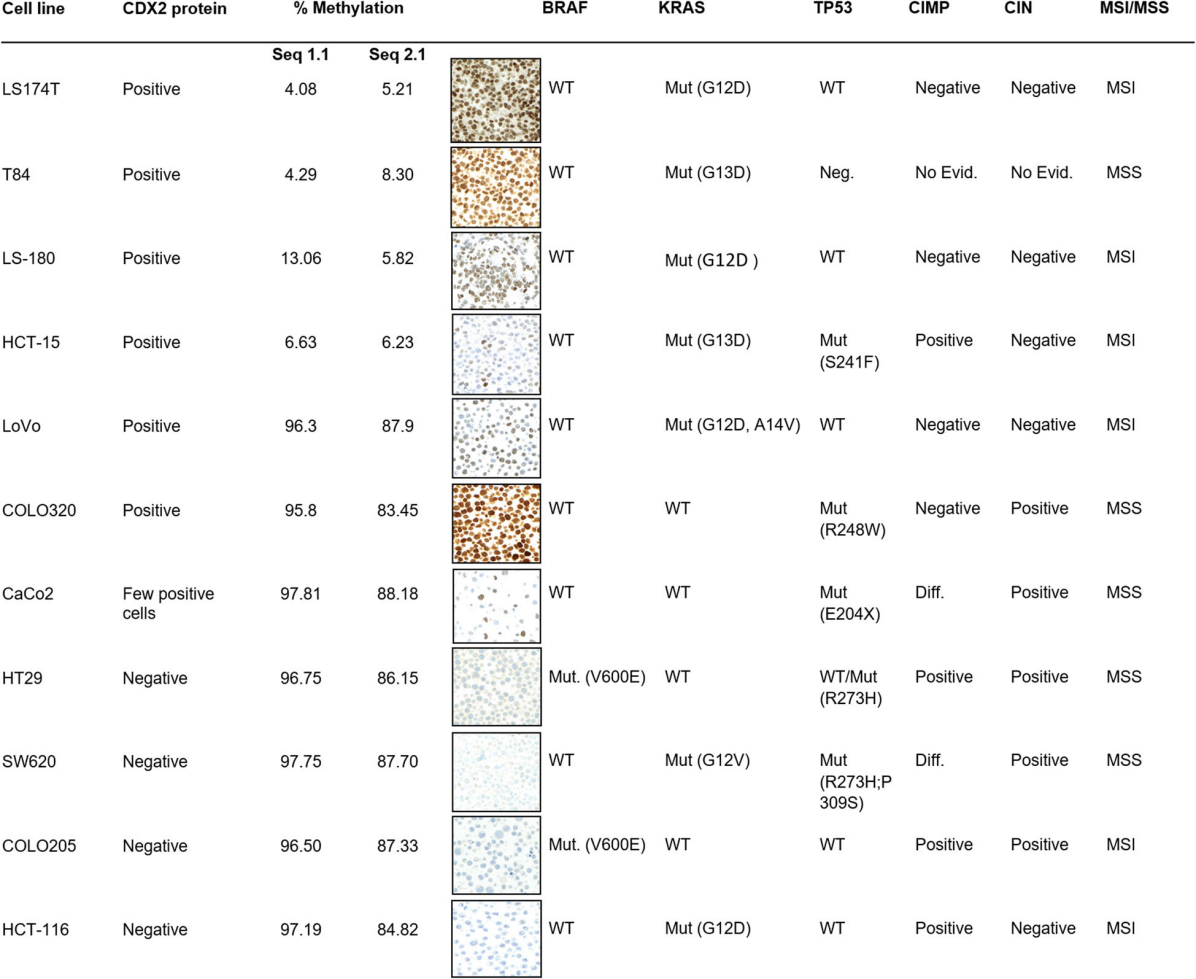
Eight colorectal cancer cell lines showing the expected inverse correlation between hypermethylation percentage at two *CDX2* promoter sequences and CDX2 protein expression

To test whether hypermethylation of *CDX2* promoter could explain mRNA and protein loss in CRCs, 11 different CRC cell lines were fixed in formalin, embedded in paraffin, and immunostained for CDX2. Diffuse, partial, or absent CDX2 expression was evaluated and correlated to the analysis of methylation status at two promoter regions. LS174T, T84, LS180, and HCT-15 were moderately to strongly positive and showed minimal methylation percentages at both sites. In contrast, HT29, SW620, COLO205, and HCT-116 showed a complete absence of CDX2 or only few CDX2-positive cells at the protein level and a high (> 80%) degree of methylation (Fig. 2). The association between higher percentage of methylation and absence of protein expression was significantly correlated (*p* = 0.0295). The remaining three cell lines, LoVo, CaCo2, and COLO320, showed no correlation between protein expression and methylation status.

### *CDX2* promoter hypermethylation and protein expression in CRCs

We selected 39 patients from cohort 1, including all tumors with BRAF mutation and MMR deficiency (*n* = 9), and performed both a *CDX2* promoter methylation analysis as well as CIMP analysis. All nine cases were CIMP-high. *CDX2* hypermethylation (> 20% methylation across all CpG sites) was found in 23 patients. Pyrosequencing results for these patients can be found in Additional file 1: Table S3. There was a striking inverse correlation between CDX2 protein and percentage of methylation, which was limited to the serrated tumor group (*r* = − 0.7). Cancers without these serrated molecular features had no correlation between CDX2 protein expression and methylation (*r* = − 0.07).

### DNA methyltransferase inhibitor (DNMTi) treatment restores CDX2 expression

**Fig. 3:**
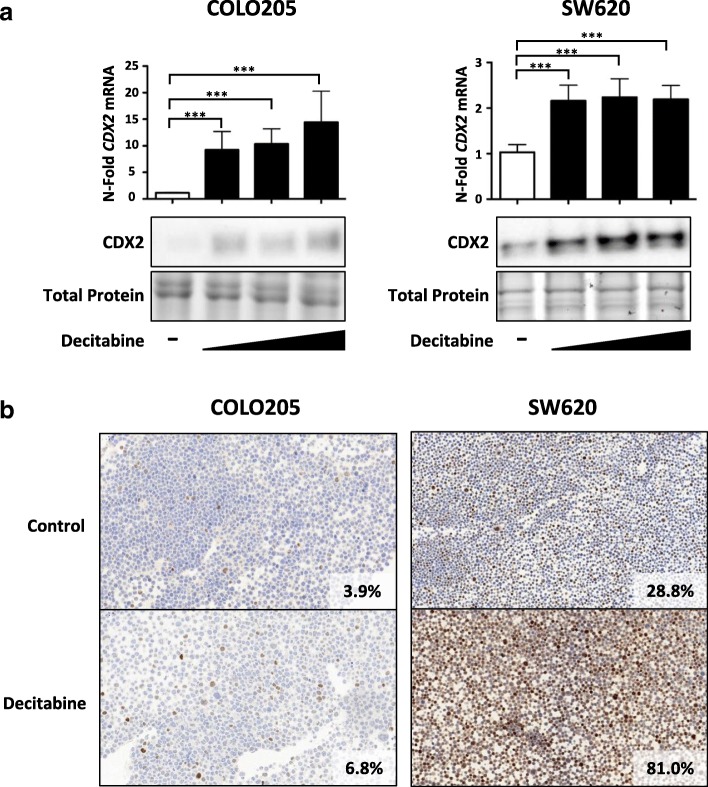
Decitabine significantly restores *CDX2* expression in CDX2-negative CRC cell lines. **a** Upper panel: qPCR analysis of CDX2-negative COLO205 and SW620 cells treated with increasing concentrations of the DNMTi decitabine (1.25 μM, 2.5 μM, 5 μM) for 48 h. Data were normalized to the *HMBS* housekeeping gene and are shown as *n*-fold regulation compared with DMSO-treated cells. MWU: ***p < 0.001, (*n* = 4) Lower panel: CDX2 Western blot analysis of cells treated as above. Total protein is shown as a loading control. **b** Immunohistochemistry (IHC) analysis of COLO205 and SW620 cells treated with 5 μM decitabine for 48 h. Quantification of CDX2 expression was done using the image analysis software QuPath

To test whether global demethylation can restore CDX2 expression, we treated two CRC cell lines (COLO205 and SW620) showing low/absent CDX2 expression with the DNMTi, decitabine. Upon 48 h treatment with decitabine, a significant 2- and 15-fold induction of *CDX2* RNA could be observed for SW620 and COLO205, respectively, with the latter showing a dose-dependency (Fig. 3a). Importantly, SW620 cells show a 4-fold higher *CDX2* basal level expression of mRNA compared to COLO205 cells.

As a control for the efficiency of the decitabine treatment, we determined the mRNA induction of hypermethylated in cancer 1 (*HIC1*) in SW620 and COLO205 cells, a gene with known promoter hypermethylation in cancer. We found a significant increase in *HIC1* mRNA levels in both cell lines upon decitabine treatment (Additional file 1: Figure S5).

On a protein level, a major induction in CDX2 protein was observed in SW620 and to a lesser extent in COLO205 cells, as seen on both Western blot and immunohistochemistry (Figs. 3b**)**.

### Combination of DNMTi and HDACi or HDAC4/5i treatment improves restoration of CDX2 expression

**Fig. 4:**
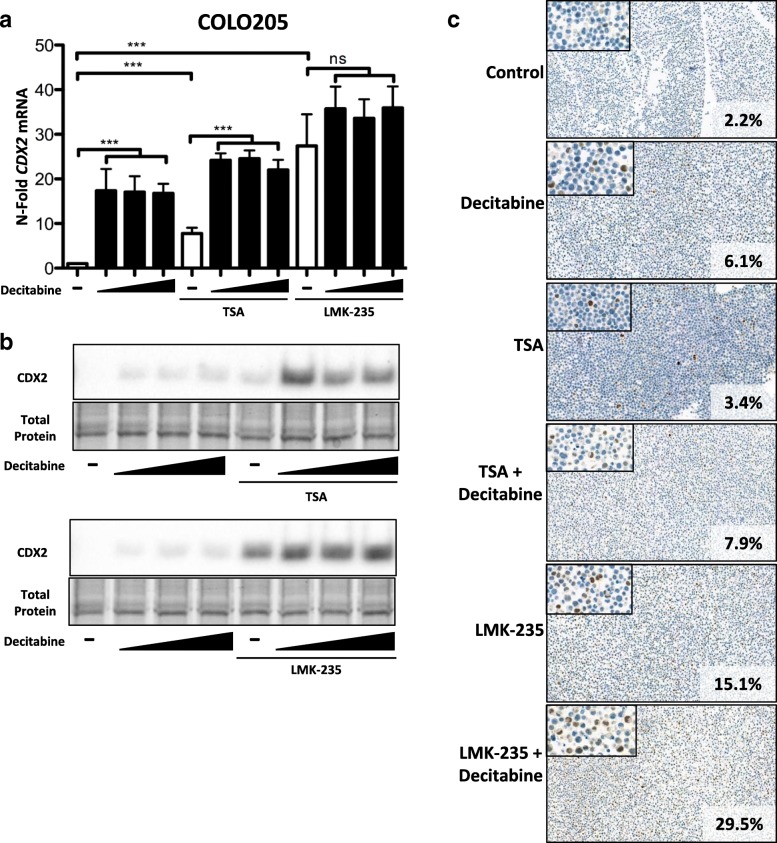
Improved CDX2 restoration in COLO205 cells upon combining DNMTi and HDACi treatment. **a** qPCR analysis of COLO205 cells treated for 48 h with DNMTi decitabine (2.5 μM, 5 μM, 10 μM) alone and in combination with the general HDACi trichostatin A (TSA; 50 nM) or the specific HDAC4/5 inhibitor LMK-235 (20 nM). Analysis as in Fig. 5a. MWU: ***p < 0.001, (*n* = 4). **b** CDX2 Western blot analysis of COLO205 cells treated as in **a**. Total protein is shown as a loading control. **c** Immunohistochemistry (IHC) analysis of COLO205 cells treated with decitabine (5 μM), TSA (50 nM), or LMK-235 (20 nM) alone or combination treatments with decitabine and TSA or LMK-235

Since DNA demethylation affects CDX2 restoration, we asked whether other epigenetic modifications, in particular, histone acetylation could have an additional impact on *CDX2* gene regulation. In a first step, we treated COLO205 and SW620 cells with a general HDACi, trichostatin A (TSA). We observed an up to 10-fold induction of *CDX2* RNA upon TSA treatment alone and an up to 23-fold induction of CDX2 RNA when combined with decitabine in COLO205 cells **(**Fig. 4a), as well as on protein level (Fig. 4b, c). This result indicates that the combination treatment of TSA and decitabine is more effective at restoring CDX2 expression than decitabine or TSA alone in COLO205. In comparison, TSA treatment alone or in combination did not have a comparable impact on CDX2 restoration in SW620 cells (Additional file 1: Figure S6). Since TSA is a general HDACi with varying specificity in HDAC inhibition, we asked whether inhibition of specific HDACs might be involved in CDX2 regulation.

We therefore treated cells with LMK-235, a specific inhibitor of HDAC4 and HDAC5. In COLO205, our results show an even more pronounced induction of CDX2, both upon single-treatment with LMK-235 (up to 25-fold) or in combination with decitabine (up to 35-fold) (Fig. 4a). These results are again underlined by increased protein expression assessed by Western blotting and immunohistochemistry (Fig. 4b, c). A similar effect can be observed in SW620 cells, namely a marked increase in both *CDX2* RNA and protein is seen upon LMK-235 treatment alone and in combination with DNMTi (Additional file 1: Figure S6).

**Fig. 5:**
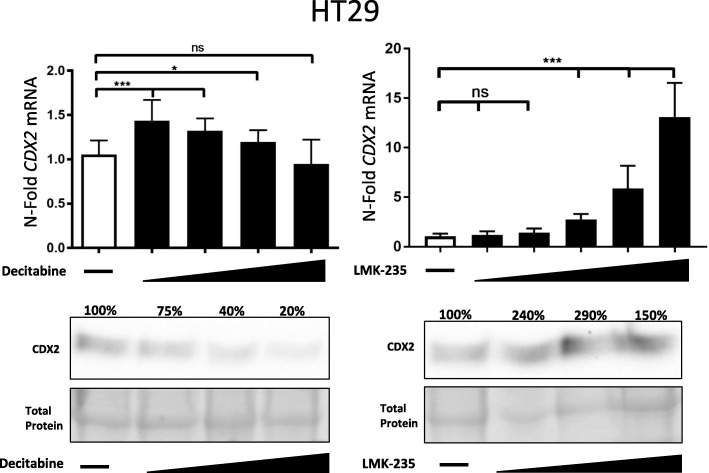
Upper panel: qPCR analysis of CDX2-negative HT29 cells treated with increasing concentrations of the DNMTi decitabine (1.25 μM, 2.5 μM, 5 μM, 10 μM) for 48 h and increasing concentrations of the HDAC4/5i LMK-235 (5 nM, 10 nM, 20 nM, 40 nM, 80 nM). Data were normalized to the HMBS housekeeping gene and are shown as *n*-fold regulation compared with DMSO-treated cells. MWU: ***p < 0.001, (*n* = 4). Lower panel: CDX2 Western blot analysis of the three highest concentrations for both compounds of HT29 cells treated as above. Total protein is shown as a loading control. Percentage indicates amount of protein normalized to respective DMSO controls

Next, we asked whether CDX2 protein restoration could be induced by LMK-235 in HT29, a cell line known to be only minimally responsive to DNMTi treatment. Indeed, upon LMK-235 treatment, HT29 cells showed a significant and dose-dependent increase of CDX2 on RNA level and remarkably on protein level as well (Fig. 5). We further observed a pronounced CDX2 induction upon LMK-235 treatment, independent of CDX2 promoter methylation status of two other cell lines, LS174T and LoVo (Additional file 1: Figure S7).

**Fig. 6:**
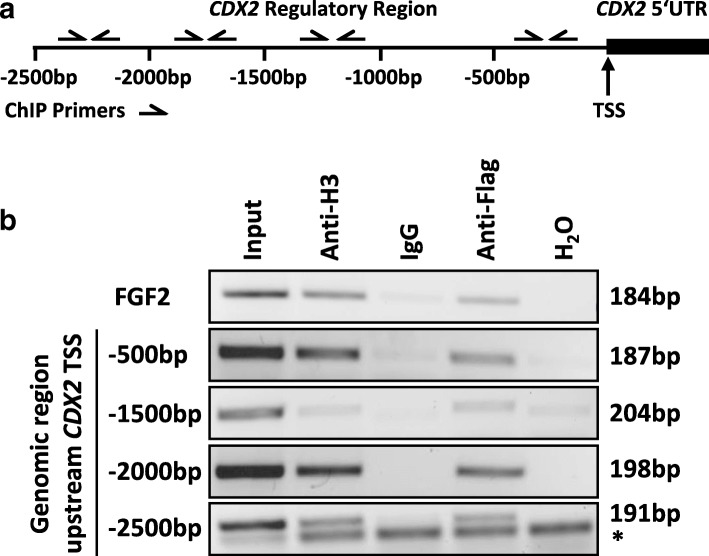
HDAC5 regulates CDX2 expression by binding to the promoter region of the *CDX2* gene. **a** Schematic representation of the genomic region upstream of the transcriptional start site (TSS) of the *CDX2* gene. ChIP primer locations are indicated by arrows. **b** In vivo binding of HDAC5 to the indicated genomic regions upstream of the *CDX2* TSS was shown by ChIP in 293T cells transfected with FLAG-tagged HDAC5 using antibodies against FLAG. Antibodies against acetyl-histone H3 and mouse IgG were used as positive and negative controls, respectively. Amplification of a genomic region in the *FGF2* gene was shown as a positive control for a genomic region bound by HDAC5. *unspecific PCR band

To test if HDAC5 localizes to the *CDX2* promoter region and is directly involved in repression *CDX2* gene expression, we performed an HDAC5 chromatin immunoprecipitation (ChIP) assay. Indeed, HDAC5 is found at a genomic region upstream of the transcriptional start site indicating direct *CDX2* repression by HDAC5 **(**Fig. 6). As a positive control for HDAC5 repressed gene expression, we amplified a genomic region of the fibroblast growth factor 2 (FGF2) gene [18].

In summary, combining decitabine with the HDAC4/5 inhibitor LMK-235 allows for improved CDX2 expression in CRC cells, particularly in cells with a low sensitivity to DNMT inhibition, whereby HDAC5 is directly involved in *CDX2* gene expression by localizing to the *CDX2* gene promoter region.

## Discussion

The novel findings of this study show that CDX2 in CRC can be regulated by either promoter methylation and more markedly by histone acetylation. In particular, treatment of cell lines by specific HDAC4 and HDAC5 inhibitor LMK-235 (with and without DNMTi) leads to a marked upregulation and re-expression of CDX2 RNA and protein, implying that both enzymes are involved in the repression of *CDX2* transcription.

CDX2 is an important prognostic factor. Its loss has been linked to more aggressive tumor features such as TNM stage, metastasis, and vessel invasion [3–5, 8, 10, 19]. Recent reports have shown that a complete loss of protein (0% staining) provides information on overall survival and chemotherapy benefit [8]. However, our study demonstrates that any loss of CDX2 can be informative, with a reduction in protein related to poorer clinical outcome in a stage-independent manner. Nolte and colleagues published a detailed analysis of CDX2 by digital image analysis on a similar number of cases, highlighting the range of possible CDX2 values expressed by CRCs [22]. They used the full information from staining intensity and percentage of positive cells and underline that any loss of protein is related to more aggressive features. Our results are in line with this observation: here, we validate the independent prognostic effect of both progressive and complete CDX2 loss of protein expression. Regardless of the large number of patients in this study (*n* = 599 for survival analysis), we cannot confirm the predictive effect of CDX2 to chemotherapy response.

Our group has previously shown that a loss of CDX2 is specific for *BRAF* mutation and for the CIMP-high phenotype and that both MSI and MSS cancers may show loss of CDX2 in this context [9, 10]. Here, we validate these findings by showing a gradual reduction in the percentage of positive cells with single (MSI or *BRAF*^*V600E*^) and double (MSI + *BRAF*^*V600E*^) alterations, findings that are in line with work from other groups [3, 23, 24]. Since the molecular characteristics of CDX2-negative tumors are predominantly those with *BRAF* mutation, CIMP, and MSI and frequently found in female patients with right-sided tumors, we hypothesized that CDX2 loss could play a functional role in tumors derived from the serrated pathway, a route of CRC development originating from the serrated adenoma. Dhir and colleagues report that CDX2 is lost in high-grade dysplastic areas of sessile serrated adenomas and may occur due to promoter hypermethylation, an observation that is directly in line with our hypothesis in cancers [14]. We therefore investigated hypermethylation as a possible mechanistic reason for the gradual loss of the CDX2 protein.

Of 11 CRC cell lines investigated, we initially selected two low-expressing cell lines (SW620 and COLO205) to evaluate whether mRNA and protein could be re-expressed upon demethylation with a broad DNMTi, decitabine, already used in clinics for treatment of some patients with myelodysplastic syndrome (MDS) and acute myeloid leukemia (AML). Here, we could demonstrate a strong re-expression of CDX2 at mRNA and protein level, thus providing a functional link between promoter methylation and protein. Although restoration of CDX2 has been previously shown for COLO205 [12], reports show that HT29 cells are not induced to express CDX2 at the protein level upon DNMTi treatment [25, 26]. The contribution of DNA promoter hypermethylation to *CDX2* gene silencing seems to vary among different CRC cells possibly reflecting a similar situation in CRC patients.

We next investigated whether HDAC inhibition could help to further restore CDX2 RNA and protein either alone or in combination with decitabine in CRC cells. We hypothesized that the more open state of chromatin coupled to demethylation would have a synergistic or additive effect on restoration of CDX2. We found that combining decitabine with a general HDACi TSA resulted in marked CDX2 induction. However, LMK-235, a specific HDAC4/5 inhibitor, had a considerably more potent effect on CDX2 restoration as compared to TSA, independent of response to decitabine treatment. Highlighting this further, protein expression of CDX2 could be restored in HT29 cells upon LMK-235 treatment, a result that is not seen upon treatment with DNMTi. Furthermore, we found direct recruitment of HDAC5 to the *CDX2* promoter region indicating that this HDAC directly represses *CDX2* gene transcription. Further, studies are needed to investigate if HDAC4 and other HDACs are also involved in repressing *CDX2* gene expression.

In human disease, it appears that loss of CDX2 is an early event in the progression of cancers via the serrated pathway [14] [27]. In our study, we can show that methylation of *CDX2* in cancers with the serrated profile have different degrees of hypermethylation that correlate with CDX2 protein in a dose-dependent manner. Few studies have examined methylation of *CDX2*. Wang and colleagues determine that the rate of hypermethylation of *CDX2* is 78.5% in colorectal cancers when compared to a normal population control (43.5%). However, this normal control group was composed of patient with colorectal polyps, likely explaining the high number of hypermethylated cases [27]. CDX2 has on the one hand been described as a tumor suppressor and its loss is associated with development of adenomas in mice [28]. On the other, it is reported as an amplified lineage-survival oncogene, sometimes amplified in CRCs and required for proliferation and survival of CRC cells [29]. Although amplification of *CDX2* was not investigated in this study, future studies may focus on this mechanism as an alternative explanation for the lack of correlation between DNA methylation and protein expression outside of the serrated tumors. Since *CDX2* mRNA (detected by ISH) and protein were so tightly linked in this study, we speculate that post-transcriptional or post-translational modification of CDX2 may play only a minor role in CRC progression.

## Conclusion

Our findings underline the independent and adverse prognostic effect of CDX2 and the involvement of epigenetic modifications in the silencing of *CDX2* gene expression, in particular of promoter methylation and histone deacetylation by HDAC4 and HDAC5. These results open a new epigenetic landscape into CDX2, which should be further investigated.

## Additional file

Additional file 1: Supplemental information. (DOCX 2156 kb)
