# Supplementary material for: CDX2 in colorectal cancer is an independent prognostic factor and regulated by promoter methylation and histone deacetylation in tumors of the serrated pathway
Source: Clin Epigenetics. 2018 Sep 26;10:120. doi: 10.1186/s13148-018-0548-2 (PMC6158822; doi:10.1186/s13148-018-0548-2)
Supplement: Supplementary file 1 — Supplemental information. (DOCX 2156 kb) [file 13148_2018_548_MOESM1_ESM.docx]

**Additional file 1**

**DNA extraction and CDX2 methylation analysis.**

Genomic DNA was extracted from selected tumoral area of FFPE tissues using QIAamp DNA FFPE Tissue Kit (Qiagen; Hilden, Germany). Bisulfite conversion and pyrosequencing were used to analyze CDX2 methylation in two different promoter regions using the EpiTect® Bisulfite Conversion Kit (Qiagen). PCR was performed from 250 ng of bisulfite converted DNA using the Pyromark PCR kit (Qiagen) with the following conditions: activation step at 95°C for 15 min and 48 cycles of denaturation 30 sec at 94 °C, annealing 30 sec at 55 °C and extension 30 seconds at 72 °C; with a final extension step for 10 min at 72 °C. Primer sequences for region 1: forward 5’-GATATTGGAGAGTATTTTAGAAATGAT AGG-3’, reverse (biotinylated) 5’-CACCTCCTAATACAAACCTTTAACA-3’ and sequencing 5’-GATAAGTGTAGGTTTTTAGG-3’. Primer sequences for region 2: Forward (biotinylated) 5’-TTAAAGGTTTGTATTAGGAGGTGA-3‘, reverse 5’-ACCAAAAAACCTAAAACTAAAAATA-3’ and sequencing 5’-ACCCCAAATCCAAAACTACCCC-3’. Universally methylated and unmethylated genomic DNA was used as positive or negative control, respectively (Epitec® PCR Control DNA; Qiagen). The PCR products were analyzed using Qiaxcel system (Qiagen). Pyrosequencing was performed on the PyroMark Q24 Instrument (Qiagen) according to the manufacturer’s instructions. The pyrosequencing results were analyzed using the PyroMark Q24 version 2.0.6 software (Qiagen).

**Table S1: Patient characteristics of Cohorts 1 and 2**

| **Clinicopathological feature** | | **Cohort 1 (n=252)** | **Cohort 2 (n=385)** |
| --- | --- | --- | --- |
|  |  | **Freq (%)** | **Freq (%)** |
| **Gender** | **Male** | 136 (54.0) | 232 (60.3) |
|  | **Female** | 116 (46.0) | 153 (39.7) |
|  |  |  |  |
| **Histological subtype** | **Adenocarcinoma** | n/a | 305 (79.2) |
|  | **Mucinous** | n/a | 44 (11.4) |
|  | **Other** | n/a | 9 (2.4) |
|  |  |  |  |
| **Tumor location** | **Left** | 39 (15.5) | 145 (37.7) |
|  | **Right** | 213 (84.5) | 120 (31.2) |
|  | **Rectum** | 0 (0.0) | 95 (24.7) |
|  |  |  |  |
| **pT** | **pT1+pT2** | 49 (19.4) | 66 (17.1) |
|  | **pT3+pT4** | 203 (80.6) | 292 (75.8)) |
|  |  |  |  |
| **pN** | **pN0** | 151 (60.2) | 175 (45.5) |
|  | **pN1-2** | 100 (39.8) | 201 (52.2) |
|  |  |  |  |
|  |  |  |  |
| **pM (or cM)** | **pM0** | 218 (86.9) | 323 (83.9) |
|  | **pM1-2** | 33 (33.1) | 61 (15.8) |
|  |  |  |  |
| **Tumor grade** | **G1-2** | 220 (87.3) | 252 (65.5) |
|  | **G3** | 32 (12.7) | 80 (20.8) |
|  |  |  |  |
| **Lymphatic invasion** | **L0** | n/a | 92 (23.9) |
|  | **L1** | n/a | 199 (51.7) |
|  |  |  |  |
| **Venous invasion** | **V0** | n/a | 132 (34.3) |
|  | **V1** | n/a | 167 (43.4) |
|  |  |  |  |
| **Perineural invasion** | **Pn0** | n/a | 194 (50.4) |
|  | **Pn1** | n/a | 53 (13.8) |
|  |  |  |  |
| **BRAF (VE1)** | **Wild-type** | 217 (86.1) | 319 (92.7) |
|  | **Mutated** | 34 (13.5) | 25 (7.3) |
|  |  |  |  |
| **MMR status** | **Deficient** | 25 (9.9) | 29 (14.4) |
|  | **Proficient** | 227 (90.1) | 172 (85.6) |
|  |  |  |  |
| **Postoperative therapy** | **None** | 154 (65.0) | 209 (54.3) |
|  | **Treated** | 83 (35.0) | 105 (27.3) |
|  |  |  |  |
| **5-year OS** | **Rate (95%CI)** | 66.6 (63-70) | 60.7 (58-63) |

n/a: not available

Table S2: Colorectal cancer cell lines and supplemented culture media

| DMEM | Dulbecco′s Modified Eagle′s Medium, D6046, Sigma |
| --- | --- |
| RPMI | RPMI-1640, R8758, Sigma |
| EMEM | Minimum Essential Medium Eagle, M4526, Sigma |
| McCoy | McCoy’s 5A Medium, M8403, Sigma |
| F12-K | Dulbecco’s Modified Eagle’s Medium/Ham’s Nutrient Mixture F12, D8437, Sigma |
| L-Glu | L-Glutamine, G7513, Sigma |
| FBS | Fetal Bovine Serum, Sigma |
| NEAA | Non-Essential Amino Acids, M7145, Sigma |
| COLO205, SW620, COLO320 | RPMI + 10% FBS |
| HT29, LS174T | DMEM + 10% FBS + 2mM L-Glu |
| HCT116 | McCoy + 10% FBS |
| T84 | DMEM + 5% FBS + 2mM L-Glu |
| LoVo | F-12K + 10% FBS |
| CaCo2 | EMEM + 20% FBS |
| LS180 | EMEM + 10% FBS + 2mM L-Glu + 1% NEAA |
| HCT15 | RPMI + 20% FBS + 2mM L-Glu |

Table S3: Analysis of CDX2 methylation patterns across two promoter regions containing 8 and 11 CpG sites respected in all 39 colorectal cancers analysed

|  | **Region 1 Meth. (%) CpG sites** | | | | | | | | | **Region 2 Meth. (%) CpG sites** | | | | | | | | | | | |
| --- | --- | --- | --- | --- | --- | --- | --- | --- | --- | --- | --- | --- | --- | --- | --- | --- | --- | --- | --- | --- | --- |
| **ID** | **1** | **2** | **3** | **4** | **5** | **6** | **7** | **8** | **Mean** | **1** | **2** | **3** | **4** | **5** | **6** | **7** | **8** | **9** | **10** | **11** | **Mean** |
| **10** | 82 | 67 | 92 | 100 | 91 | 95 | 67 | 100 | 87 | 75 | 67 | 75 | 77 | 72 | 75 | 67 | 67 | 72 | 62 | 75 | 71 |
| **15** |  |  |  |  |  |  |  |  |  | 6 | 9 | 10 | 11 | 5 | 6 | 4 | 4 | 5 | 5 | 5 | 6 |
| **16** |  |  |  |  |  |  |  |  |  | 66 | 62 | 67 | 74 | 63 | 72 | 60 | 59 | 66 | 66 | 69 | 66 |
| **20** | 0 | 0 | 3 | 0 | 0 | 3 | 0 | 0 | 1 | 6 | 3 | 5 | 6 | 8 | 10 | 15 | 3 | 5 | 9 | 3 | 7 |
| **31** | 79 | 66 | 91 | 100 | 57 | 94 | 95 | 100 | 85 | 89 | 81 | 89 | 90 | 85 | 92 | 80 | 80 | 86 | 87 | 91 | 86 |
| **38** | 28 | 61 | 95 | 97 | 75 | 96 | 62 | 97 | 76 | 97 | 88 | 96 | 96 | 74 | 89 | 83 | 85 | 90 | 93 |  | 89 |
| **40** | 5 | 2 | 8 | 3 | 2 | 9 | 3 | 14 | 6 |  |  |  |  |  |  |  |  |  |  |  |  |
| **44** | 2 | 1 | 13 | 4 | 1 | 3 | 2 | 2 | 3 | 16 | 8 | 17 | 32 | 9 | 14 | 18 | 24 | 16 | 11 | 13 | 16 |
| **47** | 2 | 1 | 5 | 2 | 1 | 13 | 6 | 4 | 4 | 9 | 3 | 7 | 8 | 2 | 2 | 8 | 9 | 2 | 2 | 2 | 5 |
| **56** | 72 | 64 | 79 | 81 | 70 | 77 | 50 | 66 | 70 | 77 | 67 | 85 | 90 | 84 | 90 | 73 | 74 | 83 | 79 | 78 | 80 |
| **60** | 2 | 1 | 2 | 2 | 1 | 2 | 2 | 2 | 2 | 15 | 14 | 14 | 15 | 15 | 19 | 13 | 13 | 15 | 13 | 27 | 16 |
| **73** | 3 | 2 | 3 | 18 | 4 | 8 | 3 | 4 | 6 | 6 | 21 | 6 | 21 | 7 | 6 | 4 | 3 | 4 | 3 | 3 | 8 |
| **74** | 74 | 69 | 70 | 84 | 65 | 74 | 72 | 70 | 72 |  |  |  |  |  |  |  |  |  |  |  |  |
| **77** | 9 | 6 | 10 | 9 | 5 | 7 | 9 | 8 | 8 | 4 | 3 | 7 | 10 | 4 | 15 | 29 | 3 | 5 | 2 | 3 | 8 |
| **79** | 39 | 33 | 50 | 51 | 34 | 43 | 38 | 49 | 42 | 41 | 33 | 25 | 45 | 24 | 29 | 36 | 27 | 23 | 25 | 27 | 31 |
| **83** | 15 | 11 | 18 | 32 | 8 | 16 | 15 | 13 | 16 | 62 | 55 | 73 | 53 | 69 | 79 | 53 | 47 | 72 | 53 | 44 | 60 |
| **84** | 84 | 71 | 90 | 100 | 90 | 95 | 70 | 100 | 87 | 93 | 85 | 92 | 90 | 90 | 93 | 70 | 73 | 86 | 82 | 87 | 85 |
| **88** | 99 | 87 | 89 | 97 | 86 | 93 | 95 | 93 | 92 |  |  |  |  |  |  |  |  |  |  |  |  |
| **99** | 26 | 20 | 32 | 34 | 24 | 35 | 32 | 25 | 29 | 33 | 24 | 27 | 41 | 23 | 30 | 25 | 18 | 24 | 29 | 26 | 27 |
| **109** | 100 | 91 | 95 | 100 | 94 | 96 | 75 | 100 | 94 | 97 | 85 | 78 | 31 | 87 | 87 | 64 | 71 | 81 | 84 | 88 | 78 |
| **116** | 62 | 63 | 64 | 67 | 60 | 67 | 68 | 67 | 65 |  |  |  |  |  |  |  |  |  |  |  |  |
| **125** | 57 | 43 | 58 | 67 | 47 | 48 | 49 | 55 | 53 | 44 | 40 | 44 | 53 | 39 | 52 | 34 | 41 | 44 | 44 | 41 | 43 |
| **130** | 84 | 58 | 95 | 100 | 85 | 97 | 78 | 100 | 87 | 82 | 79 | 96 | 97 | 87 | 97 | 84 | 81 | 94 | 90 | 97 | 90 |
| **149** | 27 | 1 | 1 | 2 | 0 | 3 | 3 | 0 | 5 |  |  |  |  |  |  |  |  |  |  |  |  |
| **157** | 22 | 8 | 11 | 9 | 4 | 18 | 23 | 11 | 13 | 49 | 28 | 57 | 48 | 37 | 14 | 41 | 37 | 51 | 38 | 42 | 40 |
| **172** | 75 | 64 | 85 | 94 | 78 | 84 | 65 | 95 | 80 | 66 | 62 | 72 | 75 | 66 | 71 | 56 | 53 | 65 | 62 | 69 | 65 |
| **175** | 59 | 56 | 60 | 59 | 49 | 55 | 58 | 48 | 56 | 45 | 43 | 46 | 50 | 49 | 43 | 36 | 40 | 44 | 40 | 45 | 44 |
| **184** | 3 | 2 | 6 | 3 | 2 | 3 | 3 | 2 | 3 | 5 | 2 | 3 | 3 | 1 | 2 | 1 | 1 | 1 | 1 | 2 | 2 |
| **187** | 68 | 65 | 73 | 64 | 65 | 68 | 73 | 65 | 68 |  |  |  |  |  |  |  |  |  |  |  |  |
| **196** | 21 | 21 | 32 | 31 | 13 | 29 | 25 | 25 | 25 | 33 | 35 | 38 | 37 | 39 | 32 | 30 | 29 | 31 | 34 | 30 | 33 |
| **197** | 4 | 2 | 1 | 4 | 2 | 6 | 10 | 4 | 4 |  |  |  |  |  |  |  |  |  |  |  |  |
| **198** | 4 | 2 | 5 | 5 | 3 | 7 | 8 | 6 | 5 | 5 | 5 | 6 | 5 | 2 | 3 | 4 | 4 | 1 | 2 | 5 | 4 |
| **202** | 3 | 2 | 2 | 11 | 2 | 4 | 9 | 2 | 4 |  |  |  |  |  |  |  |  |  |  |  |  |
| **211** | 2 | 2 | 2 | 2 | 1 | 3 | 2 | 2 | 2 | 6 | 3 | 5 | 14 | 2 | 2 | 5 | 2 | 7 | 8 | 9 | 6 |
| **214** | 54 | 55 | 40 | 53 | 49 | 52 | 38 | 56 | 50 |  |  |  |  |  |  |  |  |  |  |  |  |
| **231** | 81 | 68 | 86 | 87 | 77 | 83 | 75 | 87 | 81 | 84 | 70 | 82 | 80 | 83 | 86 | 68 | 68 | 81 | 80 | 85 | 79 |
| **241** | 5 | 1 | 2 | 2 | 0 | 5 | 2 | 4 | 3 | 9 | 8 | 10 | 12 | 3 | 8 | 7 | 5 | 8 | 7 | 5 | 7 |
| **242** | 26 | 26 | 29 | 30 | 20 | 32 | 35 | 8 | 26 |  |  |  |  |  |  |  |  |  |  |  |  |
| **253** |  |  |  |  |  |  |  |  |  | 69 | 58 | 66 | 68 | 66 | 67 | 56 | 53 | 65 | 68 | 71 | 64 |

**
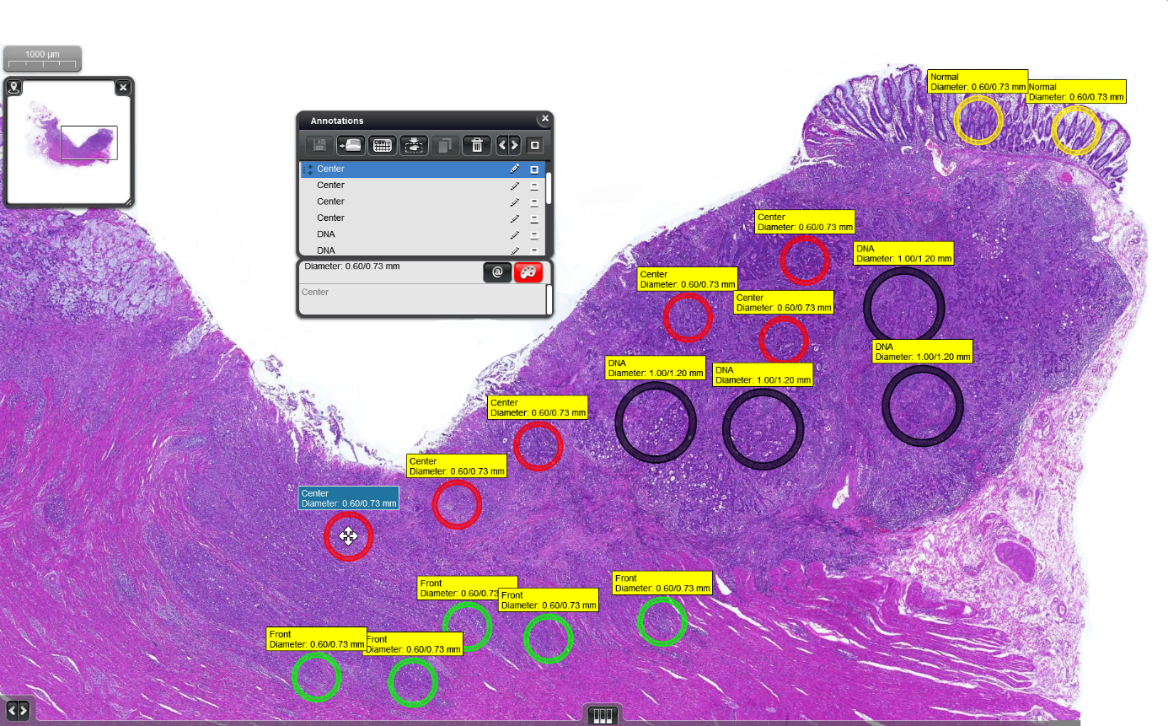
**

**Figure S1**: **Next-generation tissue microarray (ngTMA®) constructed by annotating the digital slide using annotation tools.** Red = tumor center, green = invasion front, yellow = normal tissue, and black = punches for DNA extraction.


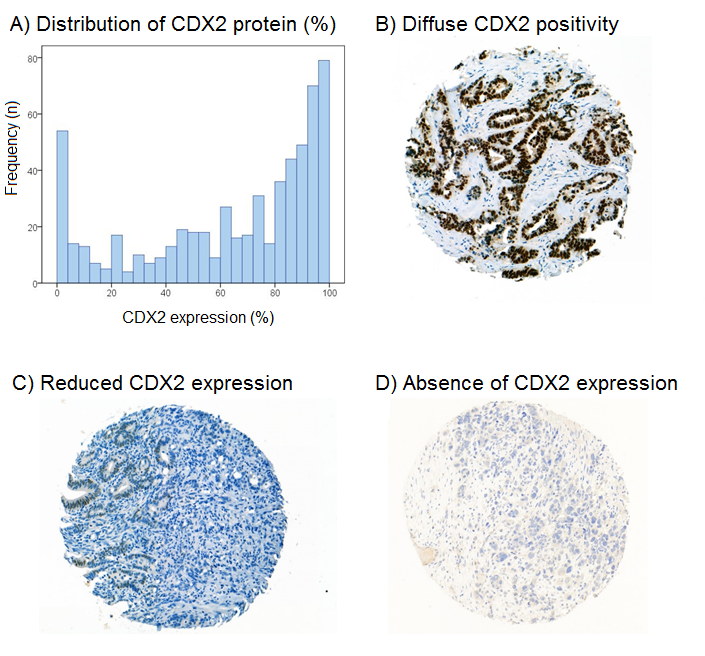


**Figure S2:** A) Distribution of CDX2 scores from 0 to 100% across both colorectal cancer cohorts and representative immunohistochemistry staining (brown) showing B) diffuse CDX2 positivity, C) reduced CDX2 protein expression and D) complete absence of CDX2 protein.

Figure S3: Correlation between CDX2 mRNA ISH scores and mean CDX2 protein expression in colorectal cancer (r =0.99; p<0.0001). Score 0 = no staining, score 1 = difficult to see under 40×, score 2 = difficult to see under 20× but easy under 40×, score 3 = difficult to see under 10× but easy under 20× and score 4 = easy to see under 10×.

**
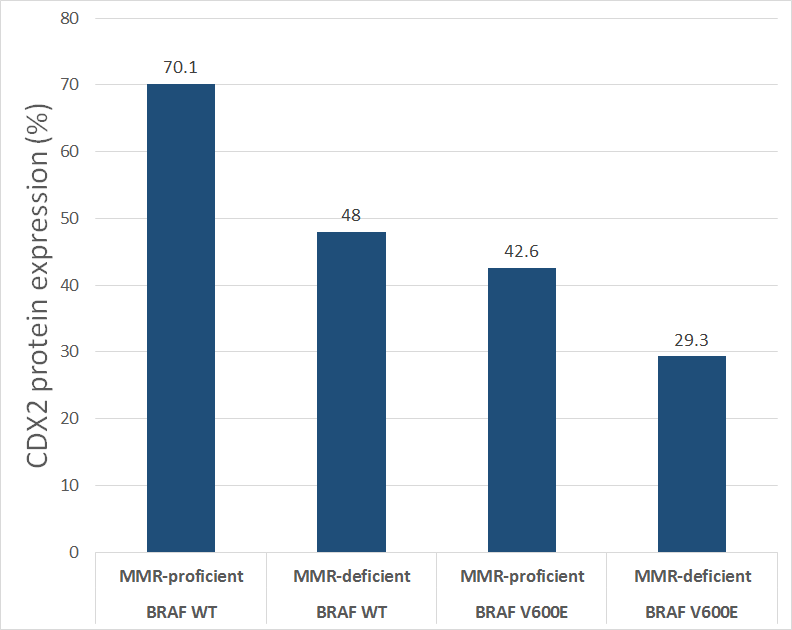
**

**Figure S4**: Changes in mean CDX2 protein expression with alterations in mismatch repair (MMR) status (proficient or deficient) and BRAF gene status (V600E or wild-type, WT)

**
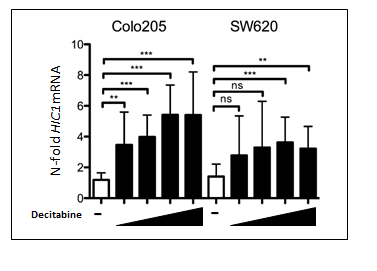
**

**Figure S5:** **Validating DNMT inhibitory activity of Decitabine.** HIC1 qPCR analysis of COLO205 and SW620 cells treated as in Figure 3A. Data were normalized to the HMBS housekeeping gene and are shown as n-fold regulation compared with DMSO treated cells.

**
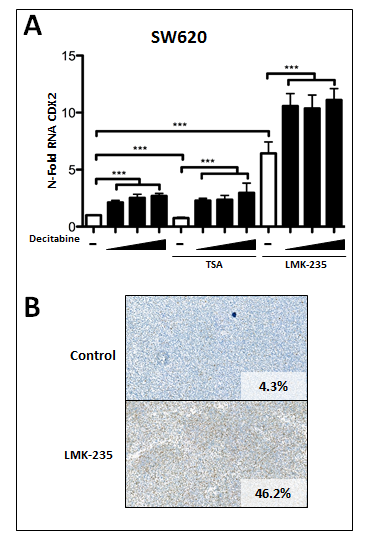
**

**Figure S6:** **Significant CDX2 restoration SW620 cells treated with a combination of DNMTi and HDACi or with LMK-235 alone**. A) qPCR analysis of SW620 cells treated for 48 hrs with DNMTi Decitabine (2.5µM, 5µM, 10µM) alone and in combination with general HDACi Trichostatin A (TSA;50nM) or the specific HDAC4/5 inhibitor LMK-235 (20nM). Analysis as in Figure 5A. MWU: ***p<0.001, (n = 4). B) Immunohistochemistry (IHC) analysis of SW620 cells treated with LMK-235 (20nM). Quantification performed using QuPath software.


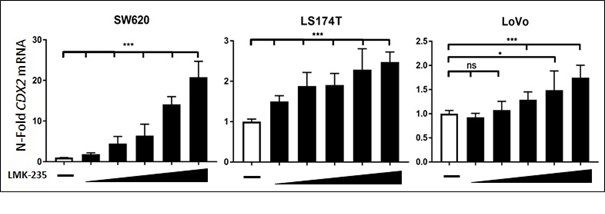


**Figure S7**: qPCR analysis of SW620 (CDX2 negative, promoter hypermethylated) and LS174T (CDX2 positive, promoter hypomethylated) and LoVo (CDX2 positive, promoter hypermethylated) cells treated with increasing concentrations of the HDAC4/5i LMK-235 (5nM, 10nM, 20nM, 40nM, 80nM). Data were normalized to the HMBS housekeeping gene and are shown as n-fold regulation compared with DMSO treated cells. MWU: ***p<0.001, (n=4)
